# Teratogenic risk of carbamazepine, levetiracetam, and lamotrigine treatment in pregnant women with epilepsy

**DOI:** 10.1590/1806-9282.20260587

**Published:** 2026-09-21

**Authors:** Guray Koc, Zeki Gokcil

**Affiliations:** 1 University of Health Sciences, Ankara Bilkent City Hospital, Department of Neurology – Ankara, Türkiye.; 2 University of Health Sciences, Gulhane Medical Faculty, Department of Neurology (Retired) – Ankara, Türkiye.

**Keywords:** Epilepsy, Pregnancy, Fetal malformations, Anticonvulsants

## Abstract

**OBJECTIVE:**

Antiseizure medications used during pregnancy may exert teratogenic effects on the developing fetus, with risks varying according to drug type, dosage, and exposure timing. While older agents such as carbamazepine are associated with known risks, newer agents including lamotrigine and levetiracetam have demonstrated more favorable safety profiles. This study aimed to evaluate pregnancy outcomes, major congenital malformation rates, and seizure frequency in pregnant women with epilepsy exposed to carbamazepine, lamotrigine, or levetiracetam as monotherapy or polytherapy, compared with an untreated control group.

**METHODS:**

This retrospective observational study included pregnant women with epilepsy who were categorized into antiseizure medication-exposed (monotherapy or polytherapy) and non-exposed groups. Clinical data, including seizure history, medication type and dosage, pregnancy outcomes, and neonatal findings, were collected. major congenital malformations were identified according to European Surveillance of Congenital Anomalies criteria.

**RESULTS:**

A total of 307 pregnancies were analyzed, resulting in 276 live births. major congenital malformation rates were 6.1% for carbamazepine, 1.7% for levetiracetam, and 3.6% for lamotrigine monotherapy. Polytherapy outcomes varied by combination, with relatively low risks observed in levetiracetam-containing regimens. Seizure frequency remained unchanged in most cases (69.6%), increased in 21%, and decreased in 9.4% of pregnancies. Preconception seizure presence significantly increased the risk of seizures during pregnancy.

**CONCLUSION:**

Levetiracetam and lamotrigine demonstrate favorable safety profiles, whereas carbamazepine carries a moderate teratogenic risk. Polytherapy risk is combination-dependent, and preconception seizure control is a key predictor of seizure outcomes during pregnancy.

## INTRODUCTION

Antiseizure medications (ASMs) can exert teratogenic and neurodevelopmental effects on the fetus. The risk of teratogenicity varies depending on the specific ASM, its dosage, and the timing of exposure during pregnancy. Some ASMs have been associated with a higher risk of congenital malformations than others. First-generation ASMs carry established teratogenic risks; notably, valproate (VPA) is not recommended in women of childbearing age due to its high risk of major congenital malformations (MCMs), including neural tube defects^1^. Carbamazepine (CBZ) has been linked with cleft lip and palate. These concerns have led to a transition toward prescribing lamotrigine (LTG) and levetiracetam (LEV), which demonstrate more favorable pregnancy safety profiles^2,3^. However, clinical experience and prescriber familiarity with newer ASMs remain more limited compared with older drugs.

Seizure control during pregnancy is another critical determinant of maternal and fetal outcomes, as seizure frequency may fluctuate throughout gestation.

This study aimed to assess pregnancy outcomes, MCM rates, and seizure frequency in pregnant women with epilepsy (PWWE) exposed to CBZ, LTG, or LEV as monotherapy or polytherapy, compared with a control group without ASM exposure.

## METHODS

This retrospective observational study included PWWE followed between June 2018 and December 2025. Participants were categorized into ASM-exposed (monotherapy or polytherapy) and non-exposed groups. The non-exposed group consisted of PWWE who were not receiving ASM treatment. For ASM-treated patients, the drug type and initial dose were recorded at baseline, and the highest dose during pregnancy was used for analysis. The introduction of a second ASM at any time during pregnancy was classified as polytherapy^2^.

Demographic and clinical variables including seizure history, ASM exposure, and seizure activity before, during, and after pregnancy were recorded. Patients were evaluated each trimester, and seizure frequency was documented at every visit. Folic acid supplementation was continued if initiated preconception or started upon pregnancy confirmation. Postnatal evaluations were performed, when feasible, at 1 month and 1 year postpartum. Infants were screened for major MCMs according to the European Surveillance of Congenital Anomalies (EUROCAT) criteria, which define MCMs as structural abnormalities of medical, functional, or cosmetic significance that require significant treatment and are identifiable at birth or within the first 6 weeks of life^4^. The observation period was extended to 1 year, consistent with International Registry of Antiepileptic Drugs and Pregnancy (EURAP) methodology^5^.

This observational study reflected routine clinical practice without interventions. To reduce retrospective bias, all eligible PWWE were included, excluding those using teratogenic drugs for non-epilepsy indications. All data were independently verified, although residual confounding cannot be excluded. Seizures were recorded for 9 months before conception, throughout pregnancy, and during the 9-month postpartum period. The average 4-week seizure frequency during the preconception period was used as baseline, and pregnancy and postpartum intervals were compared accordingly; any increase from baseline was classified as increased seizure frequency. Due to the difficulty in reliably measuring myoclonic jerks, they were not included in the analysis^6^.

### Data analysis

Statistical analyses were performed using International Business Machines Statistical Package for the Social Sciences (IBM SPSS) Statistics version 31.0 (IBM Corp., Armonk, NY, USA). Categorical variables were compared using the chi-square test, whereas non-normally distributed continuous variables were analyzed using the Kruskal-Wallis test followed by Bonferroni-adjusted pairwise comparisons. Malformation rates were determined by dividing the number of affected live births and pregnancy losses by the total number of live births and pregnancy losses. A threshold of p<0.05 was used to indicate statistical significance^2^. Maternal age, epilepsy type, preconception seizure activity, ASM regimen (no medication, monotherapy, or polytherapy), and the highest ASM dose during pregnancy were evaluated as possible confounding variables. To control for these factors, multivariable logistic regression models were applied when assessing the association between ASM exposure and MCMs, as well as seizure outcomes. Adjusted odds ratios with 95%CIs were calculated.

### Ethical considerations

This retrospective study was conducted in accordance with the Declaration of Helsinki and has been approved by the Ankara Bilkent City Hospital's Ethics Committee (approval date/number: 31.12.2025/TABED1-25-1954).

## RESULTS

Overall, 307 pregnancies in 272 women with epilepsy were retrospectively evaluated. Of the 307 pregnancies, 24 resulted in spontaneous abortion, 6 resulted in curettage, and 1 pregnancy was terminated because of meningomyelocele. A total of 276 pregnancies resulted in live birth. The mean maternal age in the cohort was 28.68±4.80 years (range 19–43). The number of pregnancies with monotherapy and polytherapy, the doses of the drugs, and the presence of MCMs are presented in Table 1. Major congenital malformations were identified in a limited number of pregnancies and were most frequently observed with CBZ at doses of 400–800 mg/day, including hypospadias, ureteral obstruction, craniosynostosis, meningomyelocele (400 mg/day) and spina bifida (800 mg/day). Single cases of hypospadias with LEV (500 mg/day) and atrial septal defect with hydronephrosis with LTG (200 mg/day) were also recorded. One case of dermal hemangioma was observed in the LEV–LTG combination group (1,500+50 mg/day) and was classified as a minor anomaly.

**Table 1:** Pregnancy outcomes according to antiseizure medication exposure.

Treatment group	N	Major congenital malformations n/N (%)	Dose (mg), median (range)
No treatment	43	0/43 (0.0)	–
CBZ	82	5/82 (6.1)	400 (150–1,000)
LEV	58	1/58 (1.7)	750 (250–2,500)
LTG	28	1/28 (3.6)	137.5 (50–450)
CBZ+LEV	31	0/31 (0.0)	400 (200–1,200)+1,000 (250–3,000)
CBZ+LTG	10	0/10 (0.0)	550 (100–1,200)+150 (100–200)
LEV+LTG	19	1/19 (5.2)	1,500 (750–3,000)+200 (50–400)
CBZ+LTG+LEV	6	0/6 (0.0)	700 (400–800)+150 (100–400)+2,000 (500–3,000)

Values are expressed as n/N (%). Doses are presented as median (range). CBZ: carbamazepine; LEV: levetiracetam; LTG: lamotrigine.

Maternal age differed significantly among groups, with untreated women being younger than those receiving monotherapy or polytherapy. Cesarean delivery rates were significantly higher in both treatment groups than in the untreated group. The proportion of focal epilepsy was highest in the polytherapy group, which differed significantly from both the untreated and monotherapy groups. In addition, seizures before pregnancy were more common in women receiving polytherapy than in those receiving monotherapy. No significant between-group differences were observed for sex, seizure frequency during pregnancy, seizure frequency postpartum, seizure occurrence during pregnancy, or postpartum seizures (Table 2).

**Table 2:** Clinical and demographic characteristics of pregnant women with epilepsy according to treatment group.

Variable		Untreated (n=43)	Monotherapy (n=167)	Polytherapy (n=66)	p-value	Post-hoc comparison
Maternal age						
	Median (IQR)	26 (23–30)	28 (25–32)	29 (19–42)	0.007	U vs. M*, U vs. P*
Gender						
	Female	46.5%	51.5%	43.9%		–
	Male	53.5%	48.5%	56.1%	0.549	
Delivery type						
	Vaginal	44.2%	23.4%	13.6%		U vs. M*, U vs. P*
	Cesarean section	55.8%	76.6%	86.4%	0.001	
Seizure type						
	Focal	37.2%	51.5%	71.2%		U vs. P*, M vs. P*
	Generalized	62.8%	48.5%	28.8%	0.001	
Seizure frequency during pregnancy						
	Decreased	11.6%	8.4%	10.6%		–
	Unchanged	58.1%	72.5%	69.7%		
	Increased	30.2%	19.2%	19.7%	0.465	
Seizure frequency postpartum						
	Decreased	16.3%	10.8%	15.2%		–
	Unchanged	60.5%	76.0%	72.7%		
	Increased	23.3%	13.2%	12.1%	0.276	
Seizure before pregnancy						
	Yes	34.9%	31.1%	54.5%		M vs. P*
	No	65.1%	68.9%	45.5%	0.004	
Seizure during pregnancy						
	Yes	39.5%	39.5%	47.0%		–
	No	60.5%	60.5%	53.0%	0.563	
Postpartum seizure						
	Yes	39.5%	29.9%	37.9%		–
	No	60.5%	70.1%	62.1%	0.329	

Post-hoc pairwise comparisons were performed using Bonferroni correction. U: untreated; M: monotherapy; P: polytherapy. Only significant pairwise comparisons are shown. IQR: interquartile range.

* Indicates a statistically significant difference between the specified groups (p ≤ 0.05).

A multivariate logistic regression analysis was conducted to identify independent predictors of congenital anomalies. The model included maternal age, seizure type, ASM dose, seizures during pregnancy, treatment type (no drug vs. monotherapy vs. polytherapy), and specific ASM combinations. The overall logistic regression model was not statistically significant (χ^2^=11.428, p=0.408), and no significant independent predictors of congenital anomalies were identified. Seizure occurrence during pregnancy showed a non-significant trend toward association (OR 4.97, p=0.082), whereas no significant associations were observed for the other variables included in the model.

**Figure 1:**
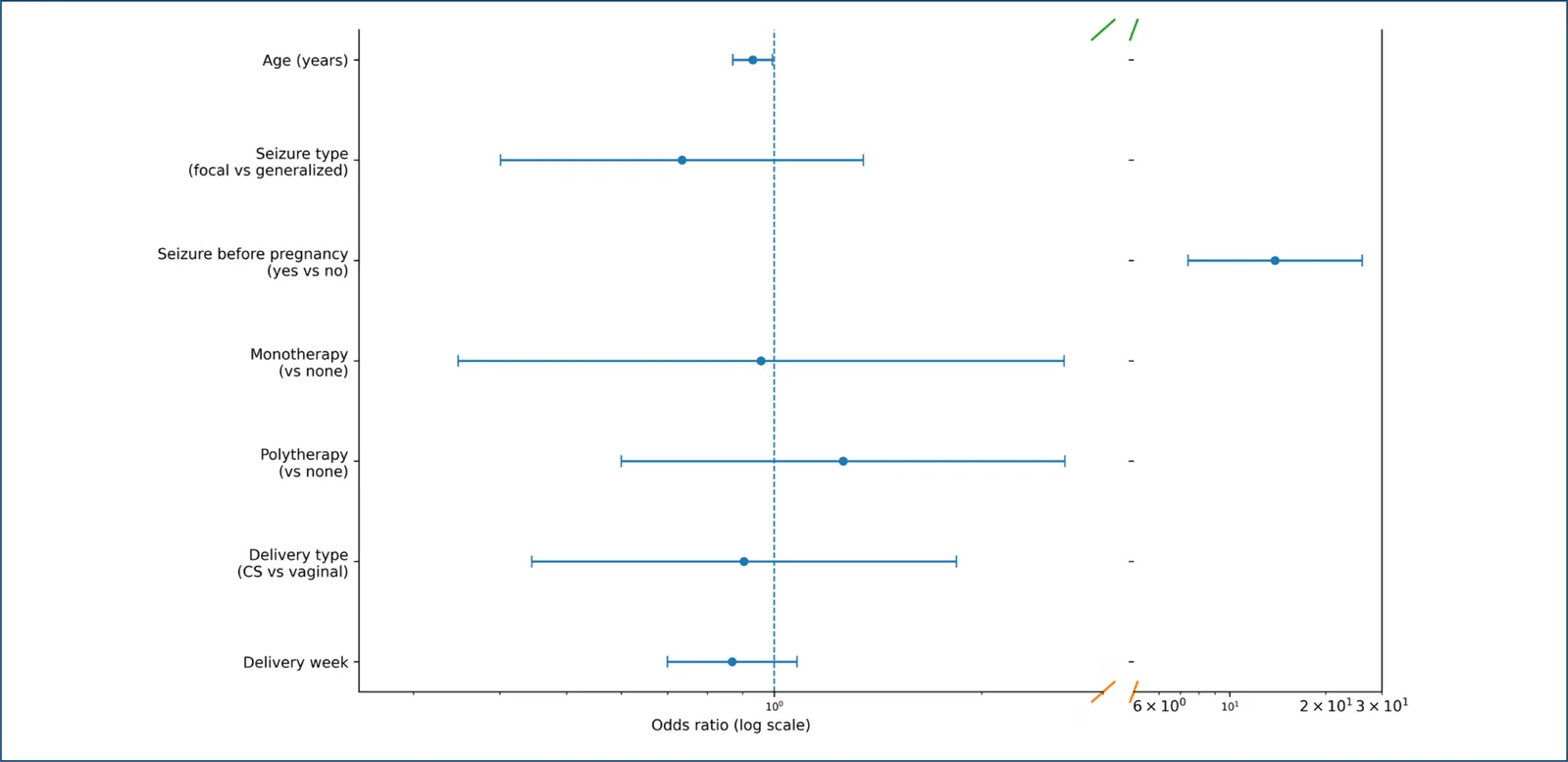
Factors associated with seizure occurrence: a forest plot analysis. Odds ratios (ORs) and 95%CIs were obtained from a multivariable logistic regression model. The x-axis is displayed on a logarithmic scale with a break to improve visualization of variables with large effect sizes.

When the seizure occurrence during pregnancy was evaluated, multivariable logistic regression analysis identified seizure history before pregnancy as a strong independent predictor of seizures during pregnancy (OR 13.87, p<0.001), while increasing maternal age was associated with a reduced risk (OR 0.93, p=0.033). The overall model was statistically significant (χ^2^=93.996, p<0.001), with good calibration (Hosmer-Lemeshow p=0.756) and acceptable discrimination (overall accuracy 77.9%) (Figure 1).

## DISCUSSION

The findings of this study reinforce the concept that teratogenic risk associated with ASM is both drug-specific and dose-dependent^7^. Although the overall risk of congenital malformations in women with epilepsy exceeds that of the general population, most pregnancies result in favorable outcomes^8^.

Previous literature has identified several potential risk factors, including higher ASM doses, polytherapy, and uncontrolled seizures during pregnancy. In our study, seizures during pregnancy showed a non-significant trend toward increased risk, yet this did not reach statistical significance in the adjusted analysis, suggesting that seizure activity alone is unlikely to exert a major teratogenic effect in the absence of other contributing factors. Seizure type was not found to be associated with MCM. We observed the highest MCM rate with CBZ, consistent with registry-based evidence indicating more favorable safety profiles for newer ASMs such as LEV and LTG^8^.

Previous studies have reported an MCM risk of approximately 4.7% with CBZ exposure, and dose-dependent teratogenic effects of CBZ have been reported in major registries^7^. Battino et al. reported that the prevalence of MCMs in PWWE treated with CBZ increased with dose, reaching 4.6% at ≤700 mg/day, 5.9% at >700–≤1,000 mg/day, and 9.1% at >1,000 mg/day^8^. In our dataset, four patients receiving 400 mg/day and one patient receiving 800 mg/day of CBZ delivered infants with MCMs. Overall, among PWWE, CBZ doses ranged from 400 to 1,200 mg/day, with an MCM rate of 6.49%, which is consistent with the literature^8^.

LEV is associated with a relatively low risk of MCMs, estimated at 0.7–2.8% in the literature, and does not appear to confer specific risks for particular malformation types^7,9^. In our study, the rate of MCMs among pregnancies exposed to LEV was 1.7%, with one case of hypospadias occurring at a daily dose of 500 mg. However, given the presence of hypospadias in first-degree relatives, the contribution of LEV remains uncertain, as familial history is associated with a 3.43-fold increased risk of MCMs^8^. Evidence indicates that LEV is associated with a lower risk of MCMs compared to other ASMs^10^. Relative to LEV, CBZ exposure was associated with a modest but statistically significant increase in MCM risk (OR 1.51, 95%CI 1.01–2.26)^7^.

LTG has demonstrated efficacy comparable to CBZ, with better tolerability in patients with focal seizures^11^. LTG has been associated with an MCM risk of approximately 2.7–3.1% and does not appear to confer specific risks for particular malformation types^7,8^. A dose-dependent teratogenic effect of LTG has been reported in the EURAP registry^9^. Comparison between LEV and LTG showed no significant difference in the risk of MCMs (OR 0.90, 95%CI 0.58–1.39)^7^. When CBZ was compared with LTG, no increased risk of MCMs was observed at CBZ doses ≤700 mg/day; however, the risk increased 1.77-fold at doses >700–≤1,000 mg/day^8^. The increased risk observed with CBZ relative to LEV, along with the absence of a significant difference between LEV and LTG, underscores the importance of ASM selection in minimizing teratogenic risk during pregnancy^12^.

Combination therapy during pregnancy may increase teratogenic risk, although this varies by ASM combination. LEV-containing regimens have generally been associated with low malformation risk, including in polytherapy. LTG is generally safe in monotherapy but may carry higher risk in combinations. CBZ demonstrates intermediate risk, varying with co-administered drugs. Collectively, these observations highlight the importance of individualized treatment planning and judicious selection of ASM regimens during pregnancy^13^. Cohen et al. performed a meta-analysis and reported that LTG-LEV duotherapy during the first trimester was associated with a 60% lower risk of MCMs compared with VPA monotherapy, supported by Class II evidence^14^. We identified one congenital malformation among pregnancies exposed to the LEV–LTG combination. These findings underscore that the safety of ASM use during pregnancy is shaped not only by the number of agents prescribed but also by the pharmacological characteristics of specific combinations. However, the heterogeneity and small size of the polytherapy groups constitute an important limitation. Since polytherapy was defined as the use of a second ASM at any time during pregnancy, differences in exposure duration, particularly during the first trimester, may have affected the findings. Therefore, polytherapy-related results should be interpreted cautiously.

The cesarean section rate in our cohort (75.7%) exceeded that reported for the general Turkish population (51.2%), which is already above the World Health Organization reference level. Notably, the polytherapy group had the highest rate (86.4%), potentially reflecting greater disease severity and a more cautious obstetric approach in these pregnancies^15^.

The primary limitation of this study is its retrospective and observational design. However, the inclusion of an internal control group, which is relatively uncommon in the literature, represents a notable strength and distinguishes this study from similar reports. In addition, the relatively low number of MCM events may have reduced the statistical power of the multivariable analyses, and comparisons among CBZ, LTG, and LEV should therefore be interpreted cautiously. The lack of data on assisted reproductive technologies (ART), which have been reported as an independent risk factor for congenital malformations, represents an additional limitation^16^.

We found that seizure history prior to pregnancy is the strongest predictor of seizure occurrence during pregnancy, aligning with evidence that highlights preconception seizure control as a crucial factor in outcomes. Increasing maternal age was associated with a modest decrease in seizure risk, while other clinical variables showed no independent association. These results emphasize the importance of optimal prepregnancy seizure management in women with epilepsy. Voinescu et al. demonstrated that seizure frequency during pregnancy is primarily predicted by preconception baseline activity^6^. Du et al. reported that seizure frequency during pregnancy does not significantly differ from prepregnancy and postpartum periods when women with epilepsy are managed in accordance with established guidelines^17^. Pennell et al. observed that seizure types did not show statistically significant differences in seizure worsening between groups^18^. We also found that seizure type was not an independent factor in seizure occurrence during pregnancy. No significant differences in seizure frequency were observed across seizure types or treatment groups before, during, or after pregnancy. Seizure control remained stable in most patients, with preconception seizure activity emerging as the strongest predictor during pregnancy, highlighting the importance of optimal control prior to conception.

## CONCLUSION

LEV and LTG appear to offer favorable safety profiles when used as monotherapy during pregnancy, whereas CBZ was associated with a higher observed MCM rate in this cohort. The impact of polytherapy appears to be dependent on the specific drug combinations used. Preconception seizure control remains a critical determinant of seizure outcomes during pregnancy. These findings emphasize the importance of individualized treatment strategies and careful ASM selection, particularly during the preconception period, in women with epilepsy.

## Data Availability

The datasets generated and/or analyzed during the current study are available from the corresponding author upon reasonable request.
